# Crystal structures of Kif2A complexed with WDR5 reveal the structural plasticity of WIN-S7 sites: Structure of WDR5 in complex with Kif2A

**DOI:** 10.3724/abbs.2025066

**Published:** 2025-04-30

**Authors:** Yang Yang, Shuting Zhang, Zhangyu Wu, Wenwen Li, Xuefang Sun, Yumi Xuan, Tianrong Hang, Li Xu, Xuemin Chen

**Affiliations:** 1 School of Life Sciences Anhui University Hefei 230601 China; 2 Center for Human Tissues and Organs Degeneration Faculty of Pharmaceutical Sciences Shenzhen Institute of Advanced Technology Chinese Academy of Sciences Shenzhen 581055 China; 3 Institute of Bio-Architecture and Bio-Interactions (IBABI) Shenzhen Medical Academy of Research and Translation (SMART) Shenzhen 518107 China

**Keywords:** WDR5, Kif2A, WIN motif, crystal structure, S7 pocket

## Abstract

Chromosome congression and spindle assembly are essential for genomic stability and proper cell division, with deficiencies in these processes linked to tumorigenesis. WD repeat-containing protein 5 (WDR5), a core component of the mixed lineage leukemia (MLL) methyltransferase complex, directly binds to kinesin family member 2A (Kif2A) to regulate these mitotic events. Despite the importance of this interaction, its structural basis for Kif2A recognition by WDR5 remains unclear. Here, we determine the crystal structure of WDR5 in complex with a Kif2A-derived peptide (residues 114–122) at a resolution of 1.85 Å. Structural analysis reveals that Kif2A engages both the WIN and S7 sites of WDR5 via Arg117 and Ser121, with Ser121 forming hydrogen bonds with WDR5 Tyr191 and Lys259, driving Tyr191 rotation and opening the S7 pocket. Additional structures of WDR5 complexed with truncated or mutated Kif2A peptides and a WDR5 Y191F variant highlight the dynamic nature of Tyr191. Notably, anti-WDR5 compounds exhibit a similar binding mode at the WDR5 WIN-S7 site. The results of mutagenesis combined with isothermal titration calorimetry (ITC) assays underscore the critical roles of Arg117 and Ser121 in mediating the binding of Kif2A to WDR5. In summary, our findings provide atomic-level insights into the molecular mechanisms underlying the non-canonical mitotic function of the MLL/WDR5 complex and highlight WIN-S7 sites as promising therapeutic targets for diseases associated with chromosomal instability, such as cancers.

## Introduction

Accurate chromosome segregation during mitosis is essential for maintaining genomic stability and preventing aneuploidy [1], a hallmark of many cancers [ 2, 3]. Central to this process is the coordination of chromosome congression and spindle assembly, which are regulated by a network of protein interactions and posttranslational modifications [ 1, 4]. Key players in this regulatory network include MLL complexes and kinesin motor proteins [ 5 – 7], both of which are essential for mitotic fidelity.

WDR5 is a highly conserved core component of MLL/SET1 methyltransferase complexes and is known for mediating histone H3 lysine 4 (H3K4) methylation, an epigenetic marker associated with active transcription [ 8– 10]. This modification is critical for regulating gene expression during development and in response to cellular signals [11]. In addition to transcriptional regulation, WDR5 also functions as a scaffold protein, facilitating the assembly of diverse protein complexes through its WD40 repeat domain, which forms a β-propeller structure [6]. WDR5 has two distinct binding pockets: the WDR5-interacting (WIN) site and the WDR5-binding motif (WBM) site [6]. The WIN site accommodates various binding partners, including H3 [ 12– 15], MLL proteins [ 16, 17], MBD3C [18], KANSL1 [19], PDPK1 [20], LANA [21] and PTEN [22], whereas the WBM site interacts with proteins such as MYC [23], RBBP5 [24], and KANSL2 [19]. WDR5 is involved in diverse cellular processes, such as promoting cellular differentiation and bone formation [25] and maintaining stem cell pluripotency and self-renewal [ 26 , 27]. Given its multifaceted roles, particularly in cancer biology, where it is frequently overexpressed and linked to oncogenesis [ 28– 30], WDR5 has emerged as an attractive drug target. Although the WIN site has been the primary focus of small-molecule inhibitor development [ 31, 32], recent studies have identified the S7 pocket as an additional binding site that enhances inhibitor binding affinity [ 20, 33– 37]. This structural pocket was initially identified through studies with OICR-9429 [ 31, 32], a small-molecule inhibitor that occupies a binding site formed by Phe133, Phe149, Pro173 and Tyr191, which is spatially distinct from the WIN site. Subsequent structural characterization by the Fesik group [34] led to the formal designation of this region as the S7 pocket, emphasizing its importance in structure-based inhibitor optimization. For example, dual-site inhibitors, such as compound 10, which targets both the WIN and S7 sites, have demonstrated potent on-target effects and preclinical efficacy in cancer models.

Emerging evidence suggests that the non-canonical role of WDR5 in mitosis is mediated by its interaction with Kif2A [7], a kinesin-13 family member that specializes in microtubule depolymerization. Kif2A plays a pivotal role in mitotic spindle assembly and chromosome segregation by regulating microtubule dynamics at plus ends, distinguishing it from cargo-transporting kinesins [ 38– 40]. Structurally, Kif2A consists of an N-terminal motor domain for microtubule binding and depolymerization, a coiled-coil stalk domain for dimerization, and a C-terminal tail domain that mediates interactions with regulatory proteins to ensure proper localization [41]. In addition to its role in mitosis, Kif2A is critical for neuronal development, where it orchestrates axon and dendrite growth [ 42, 43]. Deficiencies in Kif2A are associated with severe neurodevelopmental pathologies, including early-onset neurodegeneration [ 44, 45], cortical malformations, and microcephaly [46], highlighting its dual importance in cell division and neurogenesis. The activity and localization of Kif2A are tightly regulated through phosphorylation by mitotic kinases, including Plk1 and Aurora A [47], and through interactions with microtubule-associated proteins, such as the nuclear mitotic apparatus (NuMA) and nucleolar and spindle-associated protein (NuSAP) [ 48, 49].

Previous studies have shown that WDR5 localizes to the midbody and regulates abscission [50]. Notably, knockdown of *WDR5* or mutations within its central arginine-binding cavity lead to spindle assembly and cytokinesis defects, culminating in multinucleated cells. More recently, Ali *et al*. [7] demonstrated that WDR5 regulates Kif2A localization during mitosis, highlighting the WDR5-Kif2A interaction as essential for mitotic fidelity. However, the structural basis for this interaction has remained elusive.

In this study, we characterized the interaction between WDR5 and Kif2A using ITC experiment and determined several high-resolution crystal structures of the WDR5-Kif2A complexes through X-ray crystallography. Structural analyses revealed that Kif2A engages both the WIN and S7 pockets of WDR5, with Arg117 occupying the WIN site and Ser121 inducing Tyr191 rotation to open the S7 pocket. Mutational studies confirmed that disrupting these key interactions (R117A or S121G mutations) either abolished or weakened WDR5-Kif2A binding. These results align with prior observations linking these mutations to impaired chromosome congression and spindle assembly [7], underscoring the critical role of this interaction in mitotic fidelity. Intriguingly, we observed that small molecules that target the WIN-S7 pockets, such as compound 6b [37], mimic the binding characteristics of Kif2A, indicating a conserved binding mechanism. Collectively, our findings not only elucidate the structural basis of the WDR5-Kif2A interaction but also reveal its therapeutic potential for treating diseases driven by chromosomal instability, including cancer. Furthermore, the dynamic plasticity of the WIN-S7 sites exemplified by Tyr191 rotation provides a structural blueprint for designing next-generation WDR5 inhibitors with improved specificity and therapeutic efficacy.

## Materials and Methods

### Protein expression and purification

The DNA fragment encoding the human WDR5 seven tandem WD-40 domain (residues 24–334) was amplified via PCR from the human brain and bone marrow cDNA library and cloned and inserted into the pGEX-4T1 vector, which was modified to include an N-terminal GST tag and a TEV cleavage site (ENLYFQ/GSHM) (where ‘/’ denotes the cleavage site). The Y191F variant of WDR5 was generated using a MutanBEST kit (TaKaRa, Dalian, China) and verified via DNA sequencing. Proteins were expressed in *Escherichia coli* BL21 (DE3) cells (Novagen, Darmstadt, Germany) cultured in Luria-Bertani medium at 37°C. Protein expression was induced with 0.2 mM isopropyl β-D-1-thiogalactopyranoside (IPTG) once the optical density at 600 nm (OD600) reached 0.8, and the cultures were shifted to 16°C for incubation for 18 h. The cells were harvested by centrifugation and resuspended in Buffer A (20 mM Tris-HCl, 1 M NaCl, pH 8.0) supplemented with DNase I (10 U) and cOmplete, EDTA-free Protease Inhibitor Cocktail (1 tablet dissolved in 1 mL of sterile water), after which they were lysed by ultrasonication. After centrifugation to remove debris, the supernatant was incubated with glutathione Sepharose resin (Cytiva, Uppsala, Sweden) for 6 h at 4°C. The resin-bound GST-tagged proteins were resuspended in 20 mL of Buffer B (20 mM Tris, 400 mM NaCl, pH 8.0). The GST tag was cleaved using ~1 mg of TEV protease (purified in-house) overnight at 4°C. The cleaved proteins were dialyzed against Buffer C (20 mM Tris, 100 mM NaCl, pH 7.2) and concentrated using a 10-kD Amicon Ultra concentrator (Millipore, Darmstadt, Germany). Further purification was performed via HiTrap SP HP cation-exchange chromatography (Cytiva) using low-salt Buffer C and high-salt Buffer D (20 mM Tris-HCl, 1 M NaCl, pH 7.2). The target peak fractions were pooled, dialyzed against Buffer E (20 mM Tris-HCl, 200 mM NaCl, pH 7.5) and concentrated for subsequent crystallization or ITC experiments.

### Peptide synthesis

Peptides were synthesized by GenScript Biotechnology Co., Ltd. (Nanjing, China), and their purity (> 95%) was confirmed via mass spectrometry. The lyophilized peptides were weighed and dissolved in Buffer E, and the pH was adjusted to 7.5 with NaOH. The sequences of the peptides are as follows: Kif2A _114–120_: GSARARP; Kif2A _114–122_: GSARARPSQ; Kif2A _114–122_ (R117A): GSAAARPSQ; and Kif2A _114–122_ (S121G): GSARARPGQ.

### Isothermal titration calorimetry (ITC) assay

ITC assays were performed on a Microcal PEAQ-ITC instrument (Malvern Panalytical, Westborough, USA) at 20°C as previously described [18]. Protein concentrations were quantified by measuring the absorbance at 280 nm and diluted to 0.05 mM with Buffer E. Peptides were prepared at approximately 1 mM in the same buffer. Each titration included an initial 1 μL injection followed by subsequent 2 μL injections, which were conducted at 4-s intervals with 120 s between injections. The data were fitted to a single binding site model via MicroCal PEAQ-ITC analysis software provided by the manufacturer and are summarized in Table 1. All the experiments were performed in duplicate.

**Table:** **Table 1** ITC results in 200 mM NaCl solution

Protein	Peptide	△H (kcal/mol)	–T△S (kcal/mol)	N	*K*_D_(μM)
WDR5	Kif2A	–15.7 ± 0.13	7.48	0.91	0.78 ± 0.05
WDR5	Kif2A ^R117A^				N.D.
WDR5	Kif2A _114–120_	–9.2 ± 0.11	2.26	1.19	6.71 ± 0.32
WDR5	Kif2A ^S121G^	–10.6 ± 0.14	3.29	0.94	3.72 ± 0.23
WDR5	Kif2A ^S121A^	–13.0 ± 0.12	5.36	0.97	2.16 ± 0.11
WDR5_Y191F	Kif2A	–9.0 ± 0.08	0.92	1.27	0.96 ± 0.07

KD, N, ΔH, –TΔS stand for dissociation constant, binding stoichiometry, binding enthalpy and entropy, respectively. Each experiment was performed in duplicate. Dissociation constants ( *K*_D_s) were from a minimum of two experiments (mean±SD). N.D., not detectable binding.

### Protein crystallization, data collection and structure determination

The WDR5 protein was concentrated to 18 mg/mL. Protein-peptide complexes were prepared by mixing proteins with peptides at a 1:3 molar ratio and incubating overnight at 4°C. The precipitated complexes were removed by centrifugation. Crystals of the WDR5-Kif2A _114–122_, WDR5 (Y191F)-Kif2A _114–122_, WDR5-Kif2A _114–120_ and WDR5-Kif2A _114–122_ (S121G) complexes were grown at 289 K via the sitting drop method with 1 μL of protein-peptide mixture mixed with 1 μL of reservoir buffer. WDR5-Kif2A _114–122_ complex crystals were grown in 0.1 M HEPES (pH 7.5) and 18% (w/v) PEG 600. WDR5 (Y191F)-Kif2A _114–122_ complex crystals were grown in 0.12 M alcohol and 0.1 M Buffer System 1 [produced by mixing 1 M imidazole and 1 M MES (acid), pH 6.5] and 30% (v/v) GOL_P4K (glycerol, PEG4000). WDR5-Kif2A _114–120_ complex crystals were grown in 0.2 M ammonium acetate and 0.1 M sodium acetate, pH 4.0, with 15% (w/v) PEG 4000. WDR5-Kif2A _114–122_ (S121G) complex crystals were grown in 0.2 M imidazole malate, pH 5.5, with 24% (w/v) PEG600.

The X-ray diffraction data of the complexes were collected on beamline BL02U1/BL10U2/BL19U1 at the Shanghai Synchrotron Radiation Facility (SSRF, Shanghai, China). The datasets were processed and scaled using the autoPROC program suite [51]. The structures were solved by molecular replacement using monomeric WDR5 (PDB code: 8WXQ) as a search model in the Phaser program [52]. Peptides were manually built in Coot [53], and refinement was performed using Refmac5 in the CCP4 package suite [54] and Phenix.refine in the Phenix package [55]. PyMOL (DeLano Scientific LLC, San Carlos, USA) was used to generate all structural figures and electrostatic surface representations. The statistics for the data collection and structural refinement of the three structures are summarized in Table 2.

**Table:** **Table 2** Data collection and refinement statistics of WDR5-Kif2A complexes

	WDR5-Kif2A _114–122_	WDR5_Y191F-Kif2A _114–122_
**PDB code** **Data Collection** Wavelength (Å)	9J20 0.9792	9JWV 0.9792
Space group	*P*2 _1_	*C*2
Cell parameters		
a, b, c (Å)	64.874, 47.15, 104.309	116.29, 47.46, 129.52
α, β, γ (°)	90, 107.468, 90	90, 113.23, 90
Resolution ^a^ (Å)	40.00–1.85 (1.95–1.85)	119.02–1.80 (1.84–1.80)
Rmerge (%)	11.5 (58.7)	11.2 (48.7)
CC1/2	0.997 (0.895)	0.985 (0.831)
I/σI	10.4 (3.2)	6.2 (2.2)
Completeness (%)	100 (100)	99.6 (99.9)
Redundancy	6.8 (6.8)	3.6 (3.5)
**Refinement**		
No. reflections used/free	51689/2534	60332/3027
Resolution (Å)	34.10–1.85	58.04–1.80
*R*_work_^b^ / *R*_free_^c^ (%)	17.10/20.63	18.00/21.06
R.m.s.deviations Bonds lengths (Å)	0.013	0.007
Bond angles (˚)	1.268	0.971
*B*-factors (Å ^2^) Protein Water	25.71 29.12	23.07 29.35
No. atoms Protein Water	4808 303	4787 395
Ramachandran plot Favored/allowed/outlier (%)	96.43/3.57/0	95.78/4.22/0
	WDR5-Kif2A _114–120_	WDR5-Kif2A _114–122_ S121G
**PDB code** **Data Collection** Wavelength (Å)	9KD4 0.9792	9KD5 0.9786
Space group	*C*2	*P*2 _1_
Cell parameters		
a, b, c (Å)	116.87, 47.27, 129.26	64.61, 47.02, 103.49
α, β, γ (°)	90, 113.57, 90	90, 107.64, 90
Resolution ^a^ (Å)	59.24–1.64 (1.73–1.64)	49.31–1.80 (1.84–1.80)
Rmerge (%)	10.9 (83.7)	11.1 (42.2)
CC1/2	0.976 (0.491)	0.993 (0.901)
I/σI	4.5 (1.9)	11.3 (3.7)
Completeness (%)	98.9 (99.7)	98.9 (99.9)
Redundancy	3.3 (2.8)	6.4 (6.2)
**Refinement**		
No. reflections used/free	78345/3904	54720/2858
Resolution (Å)	51.19–1.64	49.31–1.80
*R*_work_^b^ / *R*_free_^c^ (%)	17.77/20.40	19.12/21.65
R.m.s.deviations Bonds lengths (Å)	0.013	0.005
Bond angles (˚)	1.295	0.963
*B*-factors (Å ^2^) Protein Water	18.30 25.11	19.77 24.96
No. atoms Protein Water	4748 293	4774 362
Ramachandran plot Favored/allowed/outlier (%)	95.44/4.56/0	95.29/4.71/0

^a^Values in parentheses are for highest-resolution shell.

^b^Rwork=∑hkl| |Fobs|−|Fcalc | |/∑hkl|Fobs|, where Fobs and Fcalc are the observed and calculated structure-factor amplitudes, respectively.

^C^Rfree is calculated in the same way as Rwork with 5% reflections, which were selected randomly from the refinement process.

## Results

### Overall structure of WDR5 in complex with Kif2A

**Figure 1:**
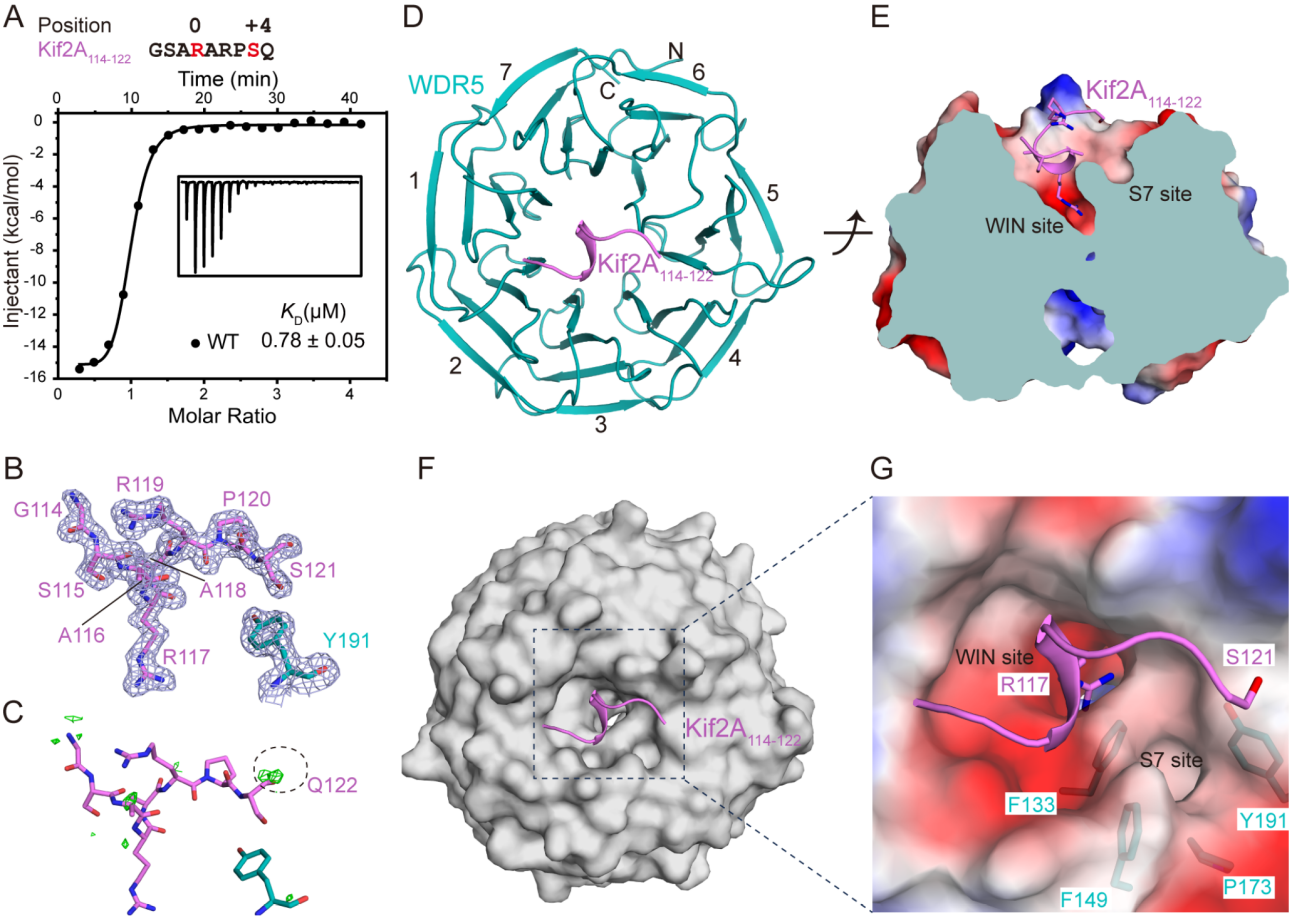
Figure 1 Overall structure of the WDR5-Kif2A _114-122_ complex (A) ITC results of WDR5 titrated with Kif2A114–122 peptide. The data were repeated in duplicate. (B,C) The 2Fo-Fc and Fo-Fc omit maps of the Kif2A114–122 peptide bound to WDR5 contoured at the 1.0 σ and 3.0 σ levels, respectively. Residues of Kif2A114–122 and WDR5 are shown as violet sticks and teal sticks, respectively. The omit map of the Kif2A Gln122 main chain is highlighted with a dashed circle. (D,E) Overview of the WDR5-Kif2A114–122 complex from two different views. WDR5 and Kif2A114–122 are colored teal and violet, respectively. (F) Surface representation of the Kif2A114–122 interacting surface of WDR5 in a view same to Figure 1D. (G) Close-up view of the WIN site and S7 site. The WDR5 surface is shown with 40% transparency. The positions of WDR5 Phe133, Phe149, Pro173 and Tyr191 are shown.

Previous studies have emphasized the importance of the WIN motif in Kif2A for its interaction with WDR5 and its role in spindle localization [7]. To quantitatively evaluate this interaction, we performed ITC assays using a synthetic peptide from Kif2A (residues 114-122: GSARARPSQ) with WDR5. The results revealed that the Kif2A peptide binds to WDR5 with a dissociation constant ( *K*_D_) of 0.78 μM ( Figure 1A and Table 1).

To further elucidate the molecular details of the Kif2A-WDR5 interaction, we crystallized the Kif2A peptide in complex with WDR5 and determined its structure at a resolution of 1.85 Å ( Table 2). Two nearly identical WDR5-Kif2A complexes (RMSD = 0.132 Å, 291 Cα atoms) were observed in one asymmetrical unit, with one model selected for subsequent structural analysis. In the final structure, residues 114–121 of Kif2A ( Figure 1B,C) and residues 31–334 of WDR5 were well traced owing to their clear electronic density map. Structural analysis revealed that the Kif2A peptide adopts an extended conformation, with a short 3 _10_ α-helix spanning the central channel on the smaller surface of the β-propeller disc of WDR5 ( Figure 1D,E). Notably, Kif2A Arg117 occupies the central channel of WDR5’s β-propeller (WIN site; Figure 1F,G), whereas Kif2A Ser121 binds to the edge of a unique hydrophobic pocket (S7 site; Figure 1G), formed by Phe133, Phe149, Pro173, and Tyr191. These findings indicated that both the WIN and S7 sites are essential for Kif2A binding.

### Role of the WDR5 WIN and S7 sites in Kif2A recognition

**Figure 2:**
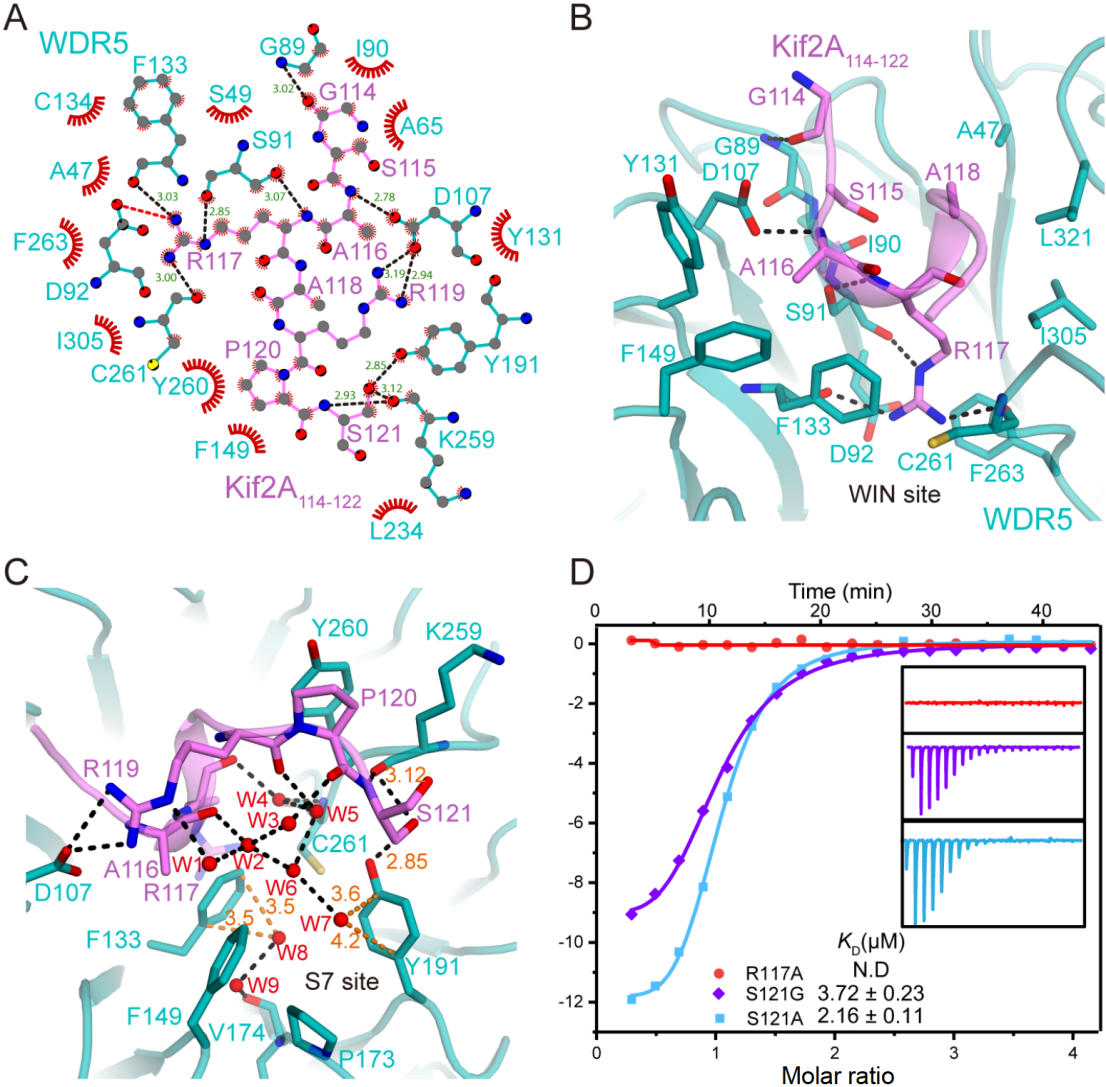
Figure 2 Binding details of WDR5 in complex with Kif2A _114-122_ (A) Schematic representations of Kif2A114–122 recognition by WDR5 were produced using the Ligplot program. Hydrogen bonds are indicated by black dashed lines whose lengths are labelled. Electrostatic interactions are shown as red dashed lines, whereas spoked arcs represent van der Waals contacts between WDR5 and Kif2A. The distances were measured in Å. (B,C) Close-up view of the binding interfaces between WDR5 and Kif2A114–122 spanning Gly114-Ala118 (B) and Arg119-Ser121 (C) Hydrogen bonds are indicated by black dashed lines. Water molecules are shown as red spheres. The distances between the water molecules and aromatic rings are labelled with orange dashed lines. (D) ITC data of WDR5 titrated with Kif2A114–122 R117A and S121G variants.

The interaction between WDR5 and Kif2A is primarily mediated by hydrogen bonds and van der Waals interactions ( Figure 2A). In the N-terminal region of Kif2A ( Figure 2B), the backbone carbonyl oxygen of Gly114 forms a hydrogen bond with the backbone amide of WDR5 Gly89. The main chain of Kif2A Ala116 forms hydrogen bonds with the side chain of WDR5 Asp107, whereas its methyl group of Ala116 engages in van der Waals contacts with the side chains of WDR5 Tyr131, Phe133, and Phe149. In the central region, Arg117 inserts into the WIN site, where hydrogen bonds stabilize its guanidinium group through interactions with the backbone carbonyls of WDR5 at Ser91, Phe133, and Cys261. Additionally, the guanidinium group is sandwiched between the aromatic rings of WDR5 Phe133 and Phe263, forming cation-π interactions. The methyl group of Ala118 makes van der Waals contacts with WDR5 Ala47 and Leu321. In the C-terminal region of Kif2A ( Figure 2C), the guanidinium group of Arg119 forms two salt bridges with WDR5 Asp107, whereas Pro120 contacts WDR5 Lys259 and Tyr260 via van der Waals contacts. Notably, the hydroxyl group of Ser121 forms hydrogen bonds with the backbone carbonyl of WDR5 Lys259 and the side chain hydroxyl of Tyr191.

In addition to the direct WDR5-Kif2A interactions, nine water molecules were identified within the WIN and S7 site regions ( Figure 2C), stabilizing the complex through an extensive hydrogen-bonding network. At the junction of the WIN and S7 sites, water molecules 1, 2, and 3 mediate the interaction between the guanidinium group of Arg119 and the backbone of Ala116 and Pro120, potentially stabilizing the Kif2A-bound state of WDR5. Water molecules 4 and 5 bridge interactions between the backbones of Kif2A Arg117 and Arg119 and WDR5 Cys261. Additionally, water molecules 7 and 8 interact directly with the aromatic rings of WDR5 Phe133 and Tyr191, stabilizing these residues through their interaction with water molecules 6 and 9, respectively.

To assess the functional importance of specific residues for WDR5-Kif2A binding, we introduced point mutations at Arg117 and Ser121, which were replaced by alanine or glycine. ITC experiments revealed that the R117A mutation completely abolished binding ( Figure 2D and Table 1), underscoring the critical role of Arg117 in WDR5 recognition. Furthermore, ITC analysis revealed that the S121A and S121G variants presented *K*_D_ values of 2.16 μM and 3.72 μM, respectively, corresponding to 1.8-fold and 3.8-fold reductions in binding affinity relative to that of wild-type Kif2A. These results confirm that Ser121 is important for stabilizing the WDR5-Kif2A interaction ( Figure 2D and Table 1). Both structural analysis and ITC experiments confirmed that Arg117, which engages the WIN site, is essential for the interaction of Kif2A with WDR5, whereas Ser121, which is associated with the S7 site, enhances binding affinity.

### Unique S7 site induced by Kif2A binding

**Figure 3:**
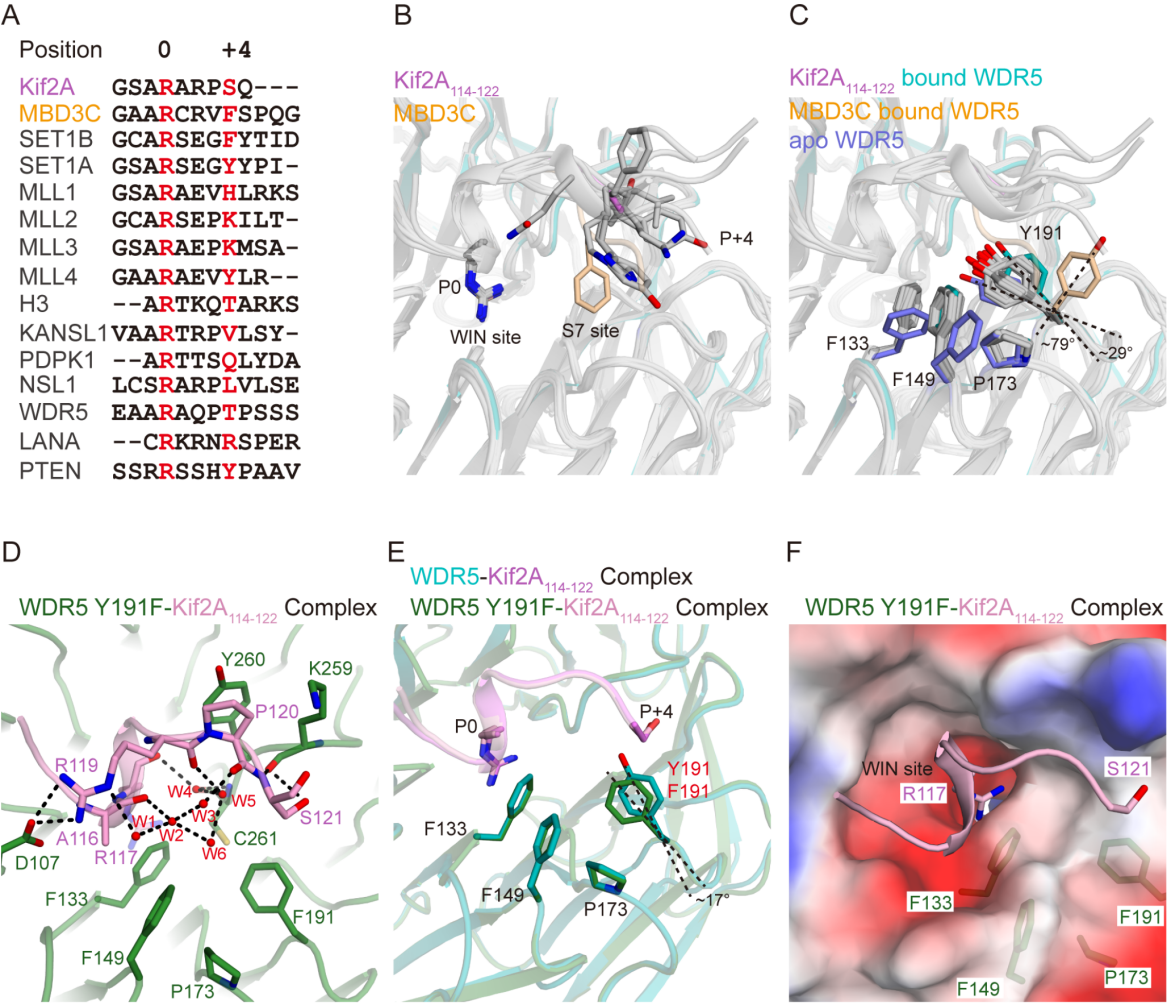
Figure 3 The formation of the WDR5 S7 site is controlled by rotation at Tyr191 (A) Sequence alignment of WIN motif-containing peptides. Residues at positions 0 and +4 are highlighted in red. These structural models were derived from the following WDR5 complexes: WDR5-MBD3C (PDB: 8WXQ), WDR5-SET1B (PDB: 8WXV), WDR5-SET1A (PDB: 3UVN), WDR5-MLL1 (PDB: 3EG6), WDR5-MLL2 (PDB: 3UVK), WDR5-MLL3 (PDB: 3UVL), WDR5-MLL4 (PDB: 3UVM), WDR5-H3 (PDB: 2H9M), WDR5-KANSL1 (PDB: 4CY1), WDR5-PDPK1 (PDB: 6WJQ), WDR5-NSL1 (PDB: 4CY3), WDR5-WDR5 (PDB: 8X3R), WDR5-LANA (PDB: 7BCY), and WDR5-PTEN (PDB: 8X3S). (B,C) Structural superposition of WDR5 in complex with the peptides shown in (A). Kif2A114–122 and MBD3C are colored violet and teal, respectively, whereas the other peptides are colored gray. (B) Residues at positions 0 and +4 are shown as sticks. (C) Apo-, Kif2A114–122- and MBD3C-bound WDR5 Tyr191 are shown as slate, teal and wheat sticks, respectively. (D) Close-up view of the binding interfaces between WDR5 Y191F and Kif2A114–122, in a view same to Figure 2C. The WDR5 Y191F variant and Kif2A114–122 are colored in forest and pink, respectively. (E) Structural superposition of the WDR5-Kif2A 114–122 and WDR5 Y191F-Kif2A114–122 complexes in a view same to (B). (F) Electrostatic potential surface of Kif2A114–122 bound to WDR5 Y191F in a view same to Figure 1F. Phe133, Phe149, Pro173 and Tyr191 are shown.

To date, many structures of WIN motif-containing peptides in complex with WDR5 have been reported [ 12– 22 ]. Sequence alignment of these peptides revealed a conserved arginine at position 0 and variable residues at position +4 ( Figure 3A). This finding correlates with the observation that all arginine residues at position 0 from these peptides are inserted into the WDR5 WIN site, whereas the variable residues at position +4 extend in various directions near the WIN site ( Figure 3B). Structural comparisons between apo WDR5 and WDR5 bound to WIN motif peptides revealed that upon binding, Phe133 and Phe149 undergo conformational rearrangements, shifting into similar positions near the WIN site ( Figure 3C). Intriguingly, Tyr191 behaves differently in response to peptide binding.

Among the WIN motif-containing peptides, Kif2A and MBD3C induce the most substantial rotation of Tyr191 in WDR5. Upon Kif2A binding, Tyr191 rotates approximately 29°, whereas MBD3C induces an even greater rotation of 79° ( Figure 3C) than does Kif2A-bound WDR5. Compared with Kif2A, the other peptides induce only minor conformational shifts in Tyr191.

We speculated that these Tyr191 conformational changes are linked to the opening of the S7 site, as previously suggested [37]. To test this hypothesis, we analyzed the electrostatic potential surface of WDR5 bound to different WIN motif-containing peptides ( Supplementary Figure S1). As anticipated, a larger pocket, termed the B site, becomes apparent in complexes where Tyr191 undergoes substantial rotation, such as with MBD3C [18]. In contrast, no discernible pocket is observed when Tyr191 undergoes only minor rotations, as observed in other peptide-bound WDR5 structures [ 12– 17, 19 – 22]. These findings suggest that the substrate-induced rotation of Tyr191 in WDR5 functions as a switch, controlling the open and closed states of the S7 site.

### Specific recognition of Tyr191 as the switch for the S7 site

As discussed, Kif2A Ser121 interacts specifically with WDR5 Tyr191, playing a critical role in WDR5-Kif2A recognition. To investigate whether this hydrogen bond interaction drives the rotation of Tyr191, we mutated WDR5 Tyr191 to phenylalanine and determined its complex structure with Kif2A ( Figure 3D and Table 2). In the final model, the WDR5 Y191F variant engages Kif2A similarly to wild-type WDR5, except the missing hydrogen bond between Kif2A Ser121 and Tyr191 ( Figure 3D and Supplementary Figure S2). Additionally, waters 7–9 are absent near Phe133. Intriguingly, the WDR5 Y191F variant exhibited a binding affinity of 0.96 μM for the Kif2A _114–122_ peptide, demonstrating comparable binding strength to that of the wild-type protein ( Table 1 and Supplementary Figure S3). Structural superposition analysis revealed that this mutation does not significantly alter the ability of WDR5 to bind to Kif2A ( Figure 3E). However, compared with the Tyr191 conformation in Kif2A-bound WDR5, the Phe191 variant rotates approximately 17° toward the conformation observed in apo WDR5, resulting in S7 site closure ( Figure 3F). These results clearly indicate that the hydrogen bond provided by Ser121 regulates the rotation of Tyr191, acting as a switch to open the S7 site.

### Residue at position +4 of Kif2A is relevant to the S7 site switch

**Figure 4:**
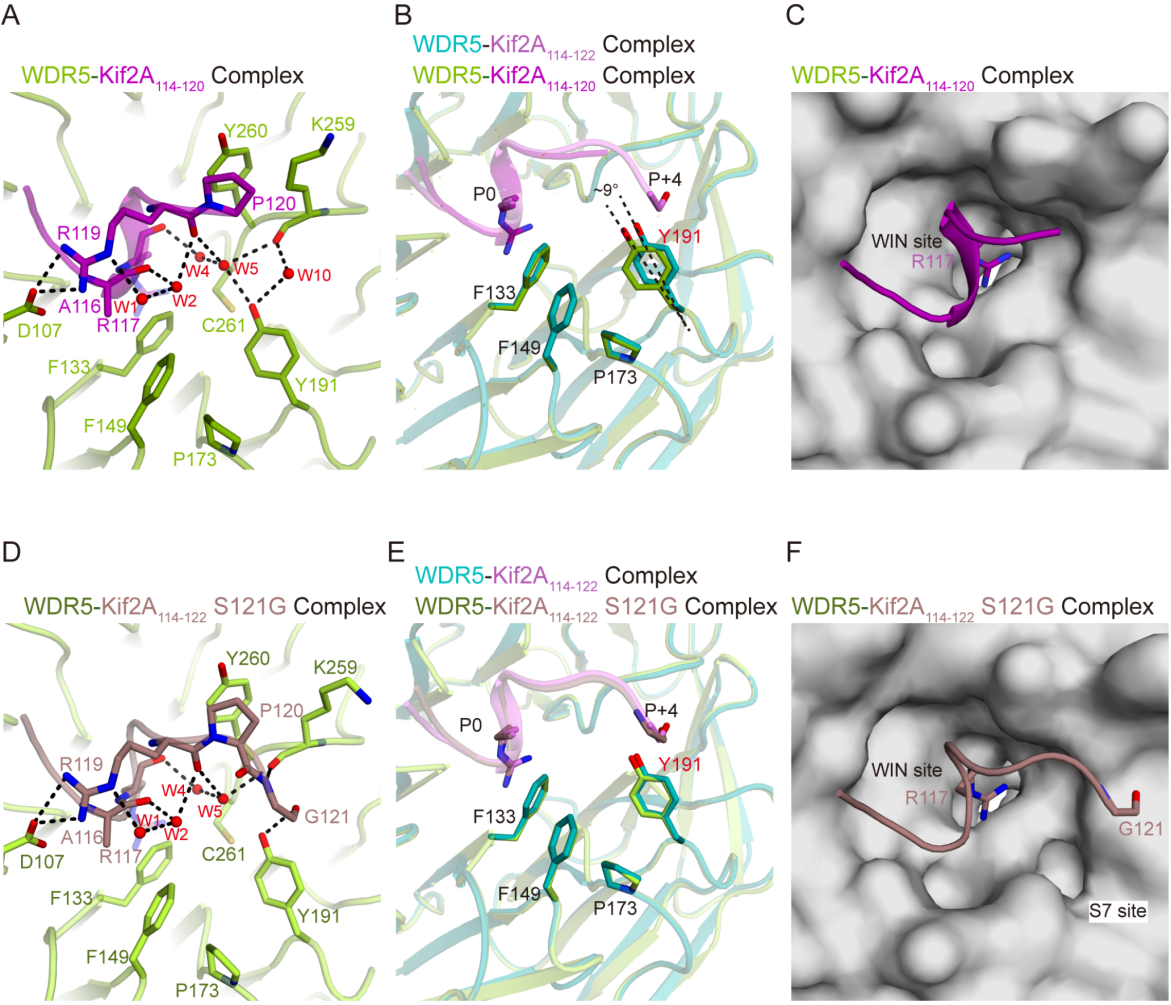
Figure 4 The residue at position +4 of Kif2A regulates the rotation of Tyr191 (A) Close-up view of the binding interfaces between WDR5 and Kif2A114–120 in a view same to Figure 2C. WDR5 and Kif2A 114–120 are colored splitpea and purple, respectively. (B) Structural superposition of the WDR5-Kif2A114–122 and WDR5-Kif2A114–120 complexes in a view same to Figure 3B. (C) Surface representation of Kif2A114–120-bound WDR5 in a view same to Figure 1F. (D) Close-up view of the binding interfaces between WDR5 and Kif2A114–122_S121G in a view same to Figure 2C. WDR5 and Kif2A114-122_S121G are colored in limon and dirty violet, respectively. (E) Structural superposition of the WDR5-Kif2A114–122 and Kif2A114–122_S121G complexes in a view same to Figure 3C. (F) Surface representation of the Kif2A114–122_S121G binding to WDR5 in a view same to Figure 1F.

To further investigate the impact of the S7 site switch induced by Kif2A, we obtained the structure of a truncated Kif2A _114–120_ complex with WDR5 ( Figure 4A and Table2). Compared with the WDR5-Kif2A _114–122_ complex, water molecules 6–9 are absent near Phe133 in the truncated complex, whereas a new water molecule (water 10) appears near Tyr191, forming a hydrogen bond with the backbone carbonyl of WDR5 Lys259. Structural superposition of WDR5 in complex with Kif2A _114–122_ and Kif2A _114–120_ revealed nearly identical conformations, except around Tyr191 ( Figure 4B). Here, Tyr191 rotates approximately 9° away from its position in the Kif2A _114-122_-bound structure, leading to S7 site closure ( Figure 4C). Additionally, the interaction of the truncated Kif2A _114-120_ peptide with WDR5 was weakened by 7.6-fold ( Supplementary Figure S3 and Table 1), indicating that Ser121 in Kif2A is crucial for its WDR5 binding.

To further explore the role of the residue at position +4, we determined the structure of WDR5 in complex with a Kif2A _114-122_ peptide carrying an S121G mutation ( Figure 4D and Table 2). Interestingly, Gly121 in Kif2A still forms a hydrogen bond with WDR5 Tyr191 via its backbone carbonyl but loses the hydrogen bond interaction with Lys259 observed in the WDR5-Kif2A_ _114–122_ complex ( Figure 4D). Structural superposition of WDR5 in complex with Kif2A _114–122_ and Kif2A _114–122__S121G revealed that the conformations of residues around the S7 site, including Phe133, Phe149, Pro173 and Tyr191, remained largely unchanged ( Figure 4E). Surface analysis revealed that the S7 site can be induced in Kif2A _114–122__S121G-bound WDR5 ( Figure 4F). These results suggested that the carbonyl group of Gly121 induces Tyr191 rotation similar to that of the hydroxyl group of Ser121. However, the S121G mutation in Kif2A reduces its binding affinity for WDR5 by 3.8-fold ( Figure 2D and Table 1), confirming that Ser121 at position +4 drives Tyr191 rotation more efficiently.

### The S7 site driven by Kif2A is accessible for compound 6b binding

**Figure 5:**
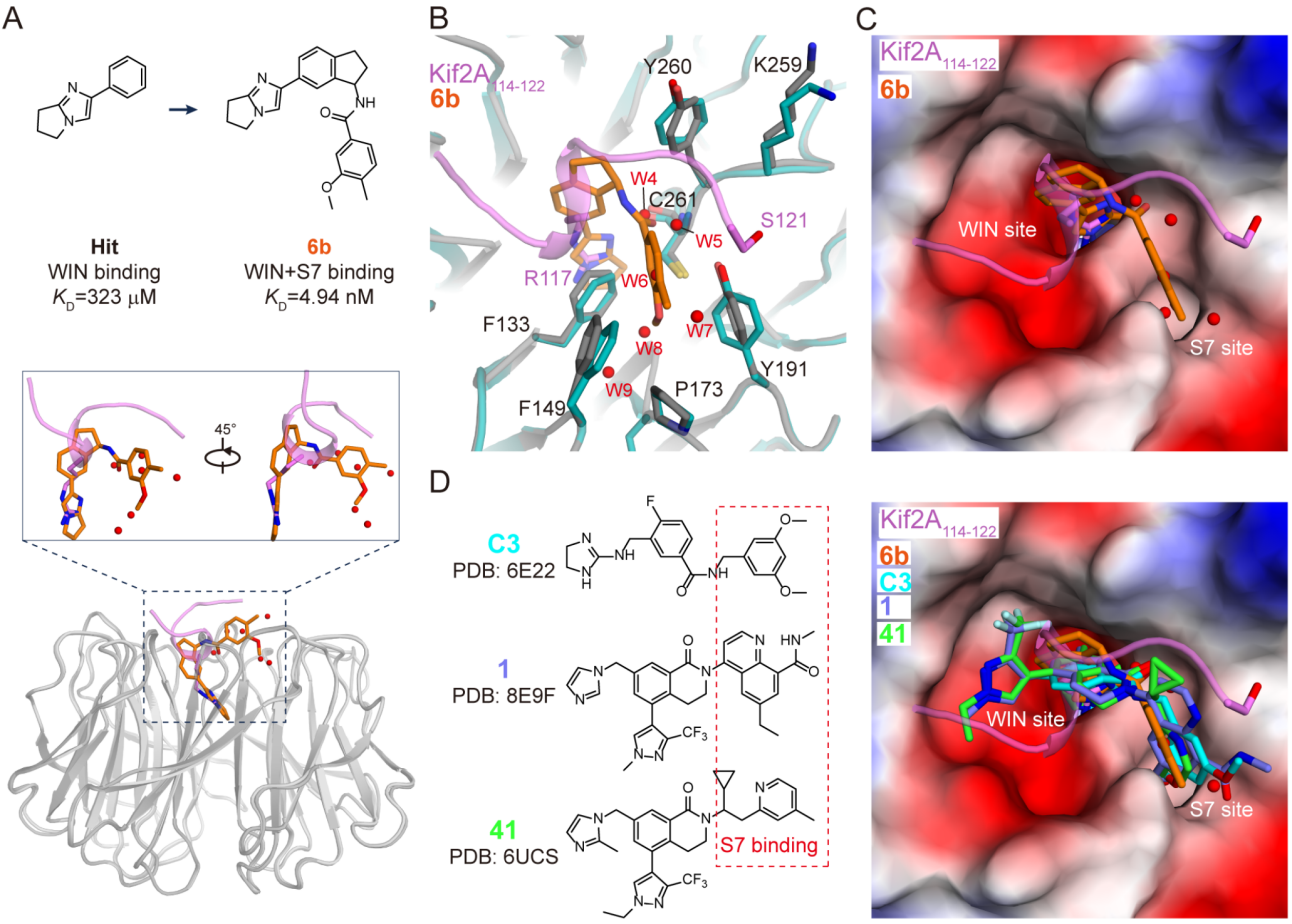
Figure 5 The S7 site is indispensable for the binding of some lead compounds (A) Structural superposition of the WDR5-6b (PDB: 6DAS) and WDR5-Kif2A114–122 complexes. Kif2A114–122 and compound 6b are colored in violet and orange, respectively. WDR5 is colored in gray. (B) Superposition of WDR5-6b and WDR5-Kif2A114–122 in a view same to Figure 2B. (C) Superposition of the WDR5-6b and WDR5-Kif2A114–122 complexes in a view same to Figure 1E. The electrostatic surface of Kif2A114–122-bound WDR5 is shown. (D) Superposition of the WDR5-Kif2A114–122, WDR5-6b, WDR5-C3, WDR5-1 and WDR5-41 complexes in a view same to Figure 1E. On the left, the chemical structural formulas of lead compounds C3, 1 and 41 are shown.

The WIN site of WDR5 has been well established as a therapeutic target in drug development [ 6 , 29]. Recent studies suggested that the adjacent S7 site also shows promise as a target for compound optimization [ 33– 37]. Wang *et al*. [37] demonstrated that dual targeting of both the WIN and S7 sites significantly enhances binding affinity (PDB: 6DAS). Specifically, a fragment that binds exclusively to the WIN site exhibited a dissociation constant ( *K*_D_) of 323 μM, whereas compound 6b, which targets both the WIN site and the S7 site, displayed a 6.5 × 10 ^4^-fold improvement in binding affinity, with a *K*_D_ value of 4.94 nM ( Figure 5A). Furthermore, compound 6b was shown to effectively displace WIN peptides from WDR5, leading to moderate growth inhibition in MLL-r-harboring cell lines [37].

Structural superposition revealed that compound 6b binds to WDR5 similarly to Kif2A. More specifically, an arginine-mimetic fragment in compound 6b occupies the WIN site, resembling the binding site of Kif2A Arg117. The aromatic tail of compound 6b extends into the S7 site, displacing the main chain of Kif2A and the water network observed in the Kif2A-WDR5 complex ( Figure 5A). The binding of compound 6b displaces the hydrogen-bonding network involving water residues 4–9 in the Kif2A-bound WDR5 structure, causing minor conformational adjustments in Phe133, Phe149, and Tyr191 ( Figure 5B). Notably, Tyr191 adopts a similar orientation in both the Kif2A- and compound 6b-bound WDR5 complexes, suggesting that the S7 site observed in the Kif2A-WDR5 complex is accessible for compound 6b binding ( Figure 5 C).

In addition to compound 6b, other high-activity lead compounds, such as C3 [56], 1 [34] and 41 [36], also target both the WIN and S7 sites ( Figure 5D). These compounds fit well into the WIN-S7 pocket of the Kif2A-bound WDR5 structure. Notably, Kif2A is the first physiological substrate identified that can induce the formation of the S7 pocket. Together, these findings indicate that Kif2A binding and small molecule interactions are complementary for understanding the dynamic interactions at WDR5 WIN-S7 sites.

### Structural plasticity of the druggable WIN-S7/B pockets

To date, the binding characteristics of various WIN motif-containing peptides and compounds within the WIN site have been extensively studied. To better understand the structural dynamics of the WIN-S7/B pockets as modulated by natural substrates and drug molecules, we closely examined these sites. Using the arginine-WDR5 complex (PDB: 6OI0), where the WIN site is occupied solely by a single arginine residue, as a reference model, we compared its structure to that of apo-WDR5. In this comparison, Tyr191 in arginine-bound WDR5 displays only a slight shift, leaving the S7 site unformed [57].

**Figure 6:**
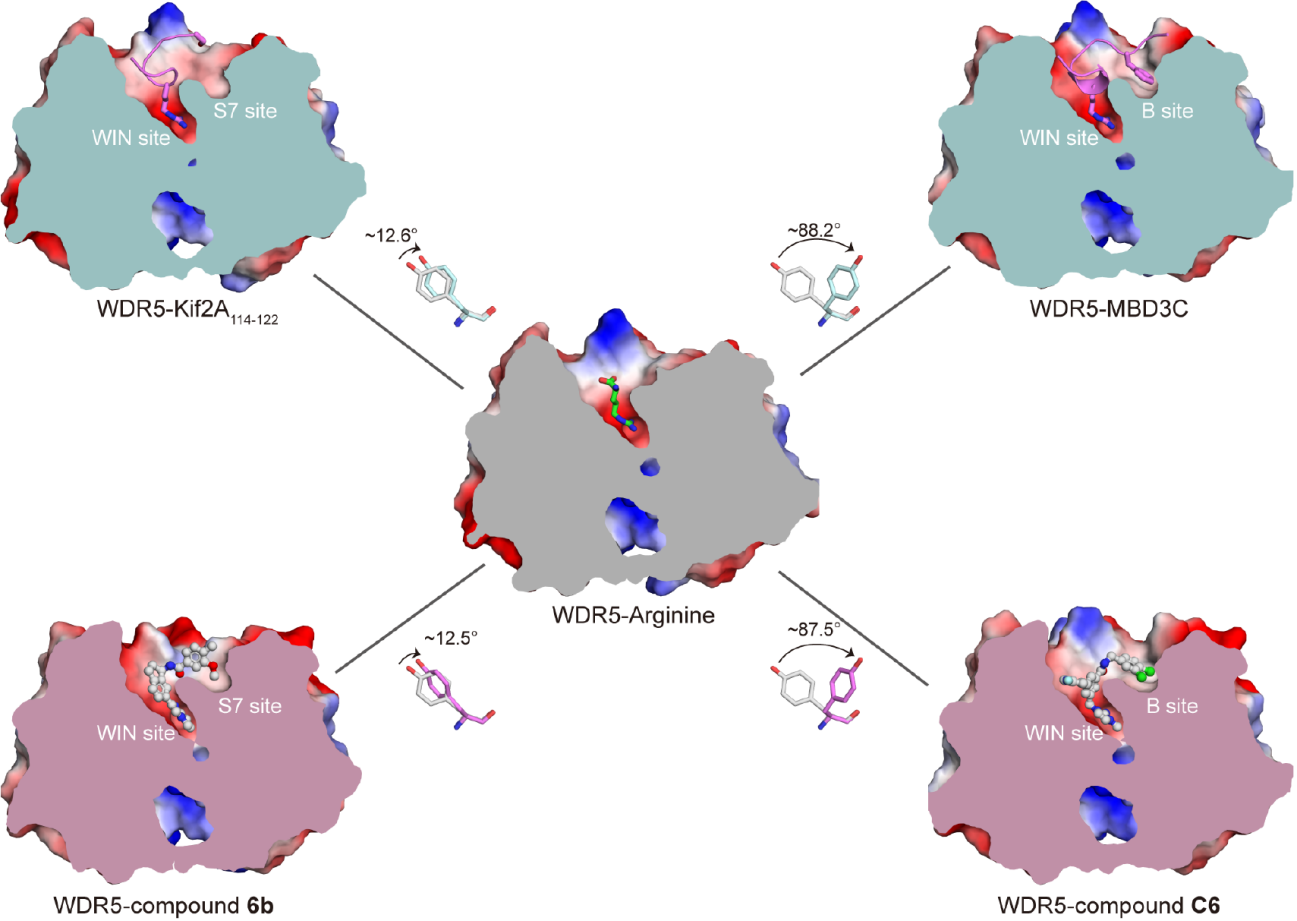
Figure 6 Tyr191 rotation is related to the WIN-S7/B dual sites The cut-open representation of the surface electrostatic potential of WDR5-Kif2A/MBD3C (PDB: 8WXQ), WDR5-arginine (PDB: 6OI0) and WDR5-6b (PDB: 6DAS)/C6 (PDB: 6E23). The interior surfaces of single arginine-bound WDR5, the peptides Kif2A/MBD3C and the compounds 6b/C6 are colored in pale cyan, gray and light pink, respectively.

In contrast, our previously determined WDR5-MBD3C complex structure shows that MBD3C binding induces an 88.2° rotation of Tyr191 ( Figure 6 and Supplementary Figure S4), facilitated by the presence of Phe47, leading to the formation of the Tyr191-related B pocket [18]. Interestingly, compound C6 induces a nearly identical 87.5° rotation, substantially increasing the binding affinity by approximately 10 ^6^-fold, which enables compound C6 to displace WDR5 from chromatin, inhibiting the proliferation of leukemia cell lines and triggering p53-dependent cell death [56].

In addition, we also investigated the interaction between Kif2A and WDR5. The structure of the complex revealed a 12.6° rotation of Tyr191 relative to its position in arginine-bound WDR5 ( Figure 6 and Supplementary Figure S4). Compared with Kif2A-bound WDR5, compound 6b induces a similar rotation at Tyr191 [37], enhancing the binding affinity of this protein approximately 6.5 × 10 ^4^-fold. These findings indicate that both the S7 and B pockets, regulated by the rotational flexibility of Tyr191, offer promising sites for small molecule binding and potential therapeutic intervention.

## Discussion

In this study, we provide structural insights into the interaction between WDR5 and Kif2A, revealing a novel mechanism by which Kif2A engages both the WIN and S7 sites on WDR5. Our structural analysis revealed that, similar to other WIN motif-containing peptides, Kif2A binds to WDR5, where the conserved Arg117 at position 0 occupies the WIN site. However, the serine at position +4 is particularly noteworthy, as it establishes key interactions with Tyr191 and Lys259, inducing rotation of Tyr191. This rotation forms the S7 pocket, a regulatory feature that distinguishes Kif2A from previously characterized WIN motif peptides, which either induce minimal Tyr191 rotation or fail to open the S7 pocket [ 12– 17, 19– 22]. While previous studies identified the S7 pocket primarily in the context of small-molecule binding [ 33– 37], our study is the first to demonstrate its induction by a physiological substrate. These findings reveal a previously unrecognized regulatory mechanism involving the dynamic behavior of Tyr191, which is pivotal in S7 pocket formation.

The functional importance of this unique binding mode was validated through ITC assays, which revealed that mutations at the key residues Arg117 and Ser121 significantly reduced the binding affinity of WDR5. These results underscore the dual importance of both the canonical WIN site interaction and the newly discovered S7 pocket in the association of Kif2A with WDR5. Importantly, this interaction is essential for the proper localization of Kif2A to the mitotic spindle, a process critical for spindle assembly and chromosome congression during mitosis [7]. Disruptions in these processes are strongly linked to aneuploidy and tumorigenesis, positioning WDR5 as a potential regulator of genomic stability during cell division.

The discovery that Kif2A, a physiological substrate, induces S7 pocket formation not only expands our understanding of the binding dynamics of WDR5 but also provides new insights for drug design. Previous studies, including those from Tansey’s group, have focused on the development of small-molecule inhibitors that exploit both the WIN and S7 pockets for therapeutic purposes, aiming to intervene in oncogenic pathways [ 33 – 37]. Our findings revealed that lead compounds such as 6b [37], C3 [56], 1 [34] and 41 [36] bind in a manner that mimics Kif2A, providing a foundation for peptide-like drug development. This offers an exciting opportunity for therapeutic strategies aimed at dual-site inhibitors that target both the WIN and S7 pockets simultaneously.

Notably, Kif2A and lead compounds activate the S7 pocket through distinct mechanisms. Lead compounds, such as 6b, forcefully open the S7 site by inserting large aromatic rings, whereas Kif2A subtly induces pocket formation via its hydroxyl group, which forms a stabilizing interaction with the hydroxyl group of Tyr191 and the carbonyl oxygen of Lys259. Interestingly, we also demonstrated that the hydroxyl group of Ser121 in Kif2A is more efficient than the carbonyl group of Gly121 at promoting S7 pocket formation (Ser121 *K*_D_ = 0.78 μM vs Gly121 *K*_D_ = 3.72 μM). This gentle yet effective mechanism of S7 pocket formation by Kif2A may provide a template for designing novel anti-WDR5 drugs with improved specificity and efficacy.

In conclusion, our study provides the first structural evidence of how Kif2A specifically engages both the WIN and S7 sites of WDR5, revealing the dynamic role of Tyr191 in regulating the S7 pocket. By elucidating the structural basis for Kif2A-induced WIN-S7 pocket formation, we lay the groundwork for further exploration of the role of WDR5 in mitosis and cancer biology. This work also establishes a framework for future studies aimed at designing more effective dual-site inhibitors targeting the WIN and S7 pockets, with potential applications in oncology and other diseases involving aberrant WDR5 activity.

## Data Availability Statement

The atomic coordinates and structural factors for the WDR5-Kif2A _114–122_ ,WDR5(Y191F)-Kif2A _114–122_, WDR5-Kif2A _114–120_ and WDR5-Kif2A _114–122_ (S121G) complexes have been deposited in the Protein Data Bank under accession numbers 9J20, 9JWV, 9KD4, and 9KD5.

## Supporting information

25102supplementary_figures

25102Tables

25105Tables

25105supplementary_figures

## Supplementary Data

Supplementary data is available at *Acta Biochimica et Biophysica Sinica* online.
