# Supplementary material for: Crystal structures of Kif2A complexed with WDR5 reveal the structural plasticity of WIN-S7 sites: Structure of WDR5 in complex with Kif2A
Source: Acta Biochim Biophys Sin (Shanghai). 2025 Apr 30;57(12):1999–2010. doi: 10.3724/abbs.2025066 (PMC12747932; doi:10.3724/abbs.2025066)
Supplement: 25105Tables [file 25105Tables.docx]

**Table 1. ITC results in 200 mM NaCl solution**

| Protein | Peptide | △H  (kcal/mol) | −T△S  (kcal/mol) | N | *K*_D_  (μM) |
| --- | --- | --- | --- | --- | --- |
| WDR5 | Kif2A | −15.7 ± 0.13 | 7.48 | 0.91 | 0.78 ± 0.05 |
| WDR5 | Kif2A ^R117A^ |  |  |  | N.D. |
| WDR5 | Kif2A _114-120_ | −9.2 ± 0.11 | 2.26 | 1.19 | 6.71 ± 0.32 |
| WDR5 | Kif2A ^S121G^ | −10.6 ± 0.14 | 3.29 | 0.94 | 3.72 ± 0.23 |
| WDR5 | Kif2A ^S121A^ | −13.0 ± 0.12 | 5.36 | 0.97 | 2.16 ± 0.11 |
| WDR5_Y191F | Kif2A | −9.0 ± 0.08 | 0.92 | 1.27 | 0.96 ± 0.07 |

*K*_D_, N, ΔH, −TΔS stand for dissociation constant, binding stoichiometry, binding enthalpy and entropy, respectively. Each experiment was performed in duplicate. Dissociation constants (*K*_D_s) were from a minimum of two experiments (mean ± SD). N.D., not detectable binding.

**Table 2. Data collection and refinement statistics of WDR5-Kif2A complexes**

|  | WDR5-Kif2A_114−122_ | | WDR5_Y191F-Kif2A_114−122_ | |  | |
| --- | --- | --- | --- | --- | --- | --- |
| **PDB code**  **Data Collection**  Wavelength (Å) | | 9J20  0.9792 | | 9JWV  0.9792 | |  |
| Space group | | *P*2_1_ | | *C*2 | |  |
| Cell parameters | |  | |  | |  |
| a, b, c (Å) | | 64.874, 47.15, 104.309 | | 116.29, 47.46, 129.52 | |  |
| α, β, γ (°) | | 90, 107.468, 90 | | 90, 113.23, 90 | |  |
| Resolution^a^  (Å) | | 40.00−1.85  (1.95−1.85) | | 119.02−1.80  (1.84−1.80) | |  |
| Rmerge (%) | | 11.5 (58.7) | | 11.2 (48.7) | |  |
| CC1/2 | | 0.997 (0.895) | | 0.985 (0.831) | |  |
| I/σI | | 10.4 (3.2) | | 6.2 (2.2) | |  |
| Completeness (%) | | 100 (100) | | 99.6 (99.9) | |  |
| Redundancy | | 6.8 (6.8) | | 3.6 (3.5) | |  |
|  | |  | |  | |  |
| **Refinement** | |  | |  | |  |
| No. reflections used/free | | 51689/2534 | | 60332/3027 | |  |
| Resolution (Å) | | 34.10−1.85 | | 58.04−1.80 | |  |
| *R*_work_^b^ /*R*_free_^c^ (%) | | 17.10/20.63 | | 18.00/21.06 | |  |
| R.m.s.deviations  Bonds lengths (Å) | | 0.013 | | 0.007 | |  |
| Bond angles (˚) | | 1.268 | | 0.971 | |  |
| *B*-factors (Å^2^)  Protein  Water | | 25.71  29.12 | | 23.07  29.35 | |  |
| No. atoms  Protein  Water | | 4808  303 | | 4787  395 | |  |
| Ramachandran plot  Favored/allowed/outlier (%) | | 96.43/3.57/0 | | 95.78/4.22/0 | |  |

|  | WDR5-Kif2A_114−120_ | WDR5-Kif2A_114−122_  S121G |
| --- | --- | --- |
| **PDB code**  **Data Collection**  Wavelength(Å) | 9KD4  0.9792 | 9KD5  0.9786 |
| Space group | *C*2 | *P*2_1_ |
| Cell parameters |  |  |
| a, b, c (Å) | 116.87, 47.27, 129.26 | 64.61, 47.02, 103.49 |
| α, β, γ (°) | 90, 113.57, 90 | 90, 107.64, 90 |
| Resolution^a^ (Å) | 59.24−1.64  (1.73−1.64) | 49.31−1.80  (1.84−1.80) |
| Rmerge (%) | 10.9 (83.7) | 11.1 (42.2) |
| CC1/2 | 0.976 (0.491) | 0.993 (0.901) |
| I/σI | 4.5 (1.9) | 11.3 (3.7) |
| Completeness (%) | 98.9 (99.7) | 98.9 (99.9) |
| Redundancy | 3.3 (2.8) | 6.4 (6.2) |
|  |  |  |
| **Refinement** |  |  |
| No. reflections used/free | 78345/3904 | 54720/2858 |
| Resolution (Å) | 51.19−1.64 | 49.31−1.80 |
| *R*_work_^b^ /*R*_free_^c^ (%) | 17.77/20.40 | 19.12/21.65 |
| R.m.s.deviations  Bonds lengths (Å) | 0.013 | 0.005 |
| Bond angles (˚) | 1.295 | 0.963 |
| *B*-factors (Å^2^)  Protein  Water | 18.30  25.11 | 19.77  24.96 |
| No. atoms  Protein  Water | 4748  293 | 4774  362 |
| Ramachandran plot  Favored/allowed/outlier (%) | 95.44/4.56/0 | 95.29/4.71/0 |

^a^Values in parentheses are for highest-resolution shell.

^b^R_work_ = ∑hkl| |Fobs|−|Fcalc | |/∑hkl|Fobs|, where Fobs and Fcalc are the observed and calculated structure-factor amplitudes, respectively.

^c^R_free_ is calculated the same as R_work_ with 5% reflections, which were selected randomly from the

refinement process
