# Supplementary material for: Crystal structures of Kif2A complexed with WDR5 reveal the structural plasticity of WIN-S7 sites: Structure of WDR5 in complex with Kif2A
Source: Acta Biochim Biophys Sin (Shanghai). 2025 Apr 30;57(12):1999–2010. doi: 10.3724/abbs.2025066 (PMC12747932; doi:10.3724/abbs.2025066)
Supplement: 25105supplementary_figures [file 25105supplementary_figures.docx]

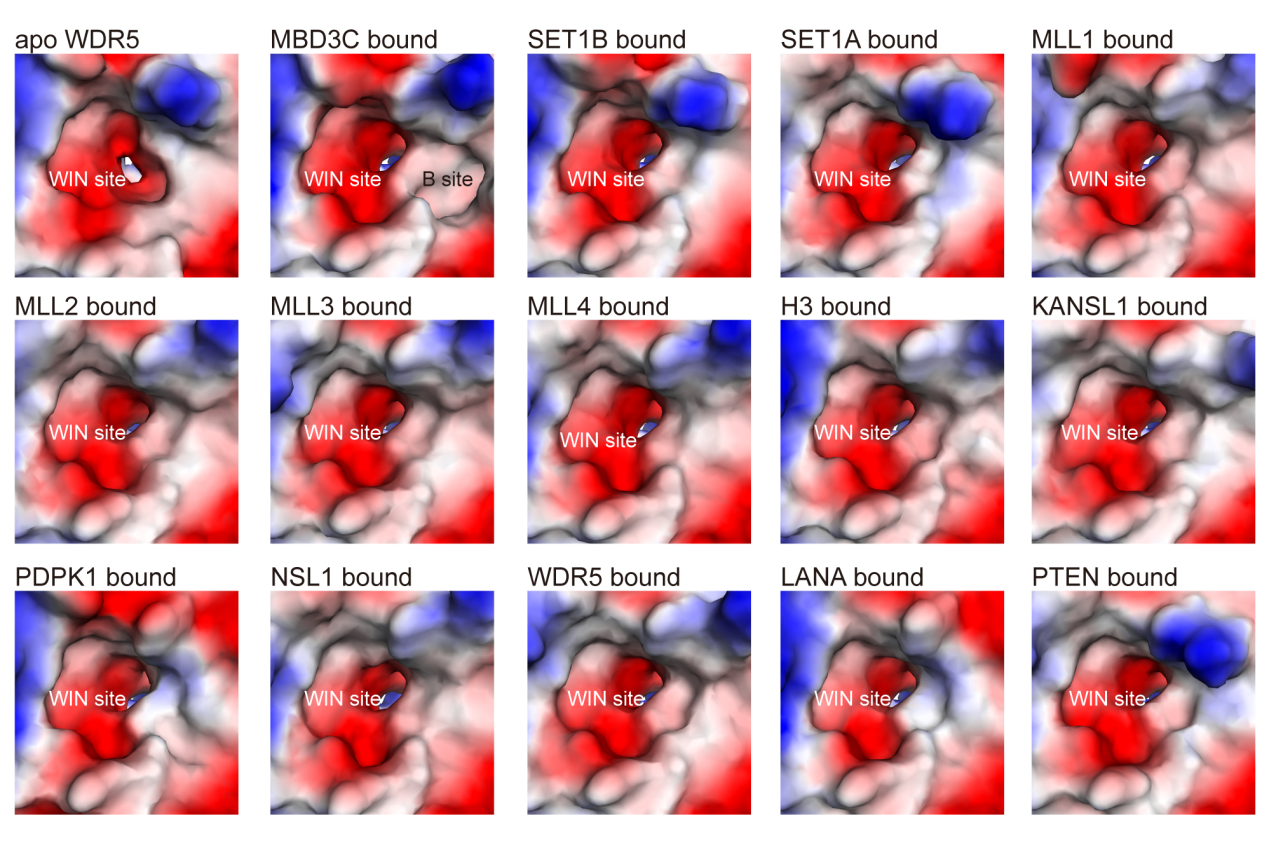


**Supplementary Figure S1.** **Electrostatic potential surface of the WDR5 bound by various WIN motif-containing peptides in a view same to** **Figure 1G** These structural models were derived from the following WDR5 complexes: apo WDR5 (PDB: 2H14), WDR5-MBD3C (PDB: 8WXQ), WDR5-SET1B (PDB: 8WXV), WDR5-SET1A (PDB: 3UVN), WDR5-MLL1 (PDB: 3EG6), WDR5-MLL2 (PDB: 3UVK), WDR5-MLL3 (PDB: 3UVL), WDR5-MLL4 (PDB: 3UVM), WDR5-H3 (PDB: 2H9M), WDR5-KANSL1 (PDB: 4CY1), WDR5-PDPK1 (PDB: 6WJQ), WDR5-NSL1 (PDB: 4CY3), WDR5-WDR5 (PDB: 8X3R), WDR5-LANA (PDB: 7BCY), and WDR5-PTEN (PDB: 8X3S).

**
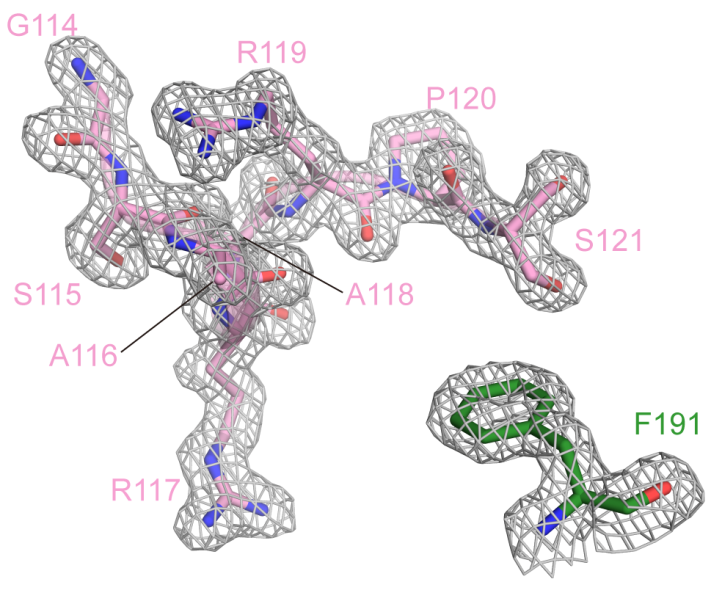
**

**Supplementary Figure S2**. **2Fo-Fc omit map of WDR5 Y191F and Kif2A114-122 peptide complex, shown in a view similar to Figure 3D**


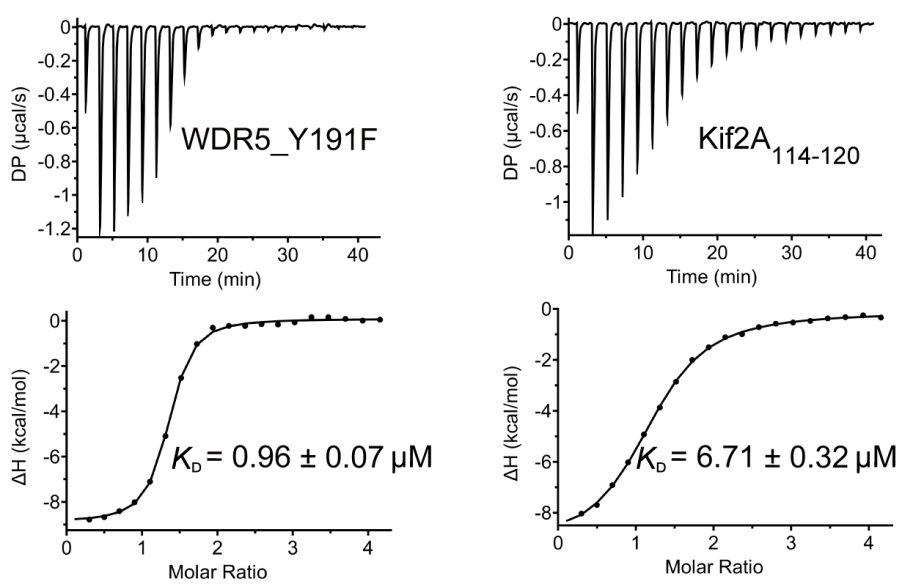


**Supplementary Figure S3**. **ITC results of the WDR5 titrated with Kif2A114-120 peptide, and WDR5_Y191F titrated with Kif2A114-122 peptide**


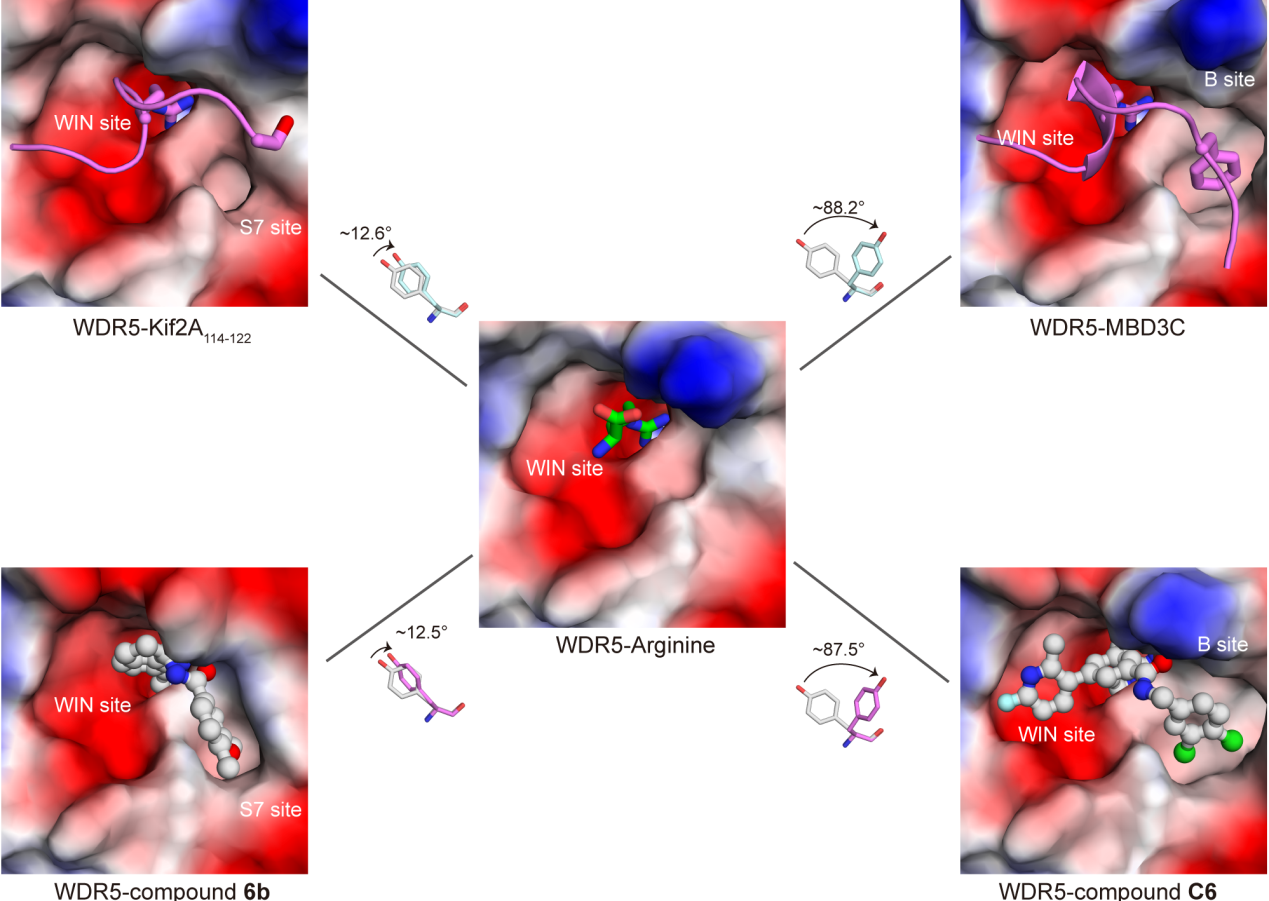


**Supplementary Figure S4. Surface electrostatic potential representation of WIN/S7/B site of Arginine, Kif2A, MBD3C, Compound 6b and compound C6 bound WDR5 in a same view**
